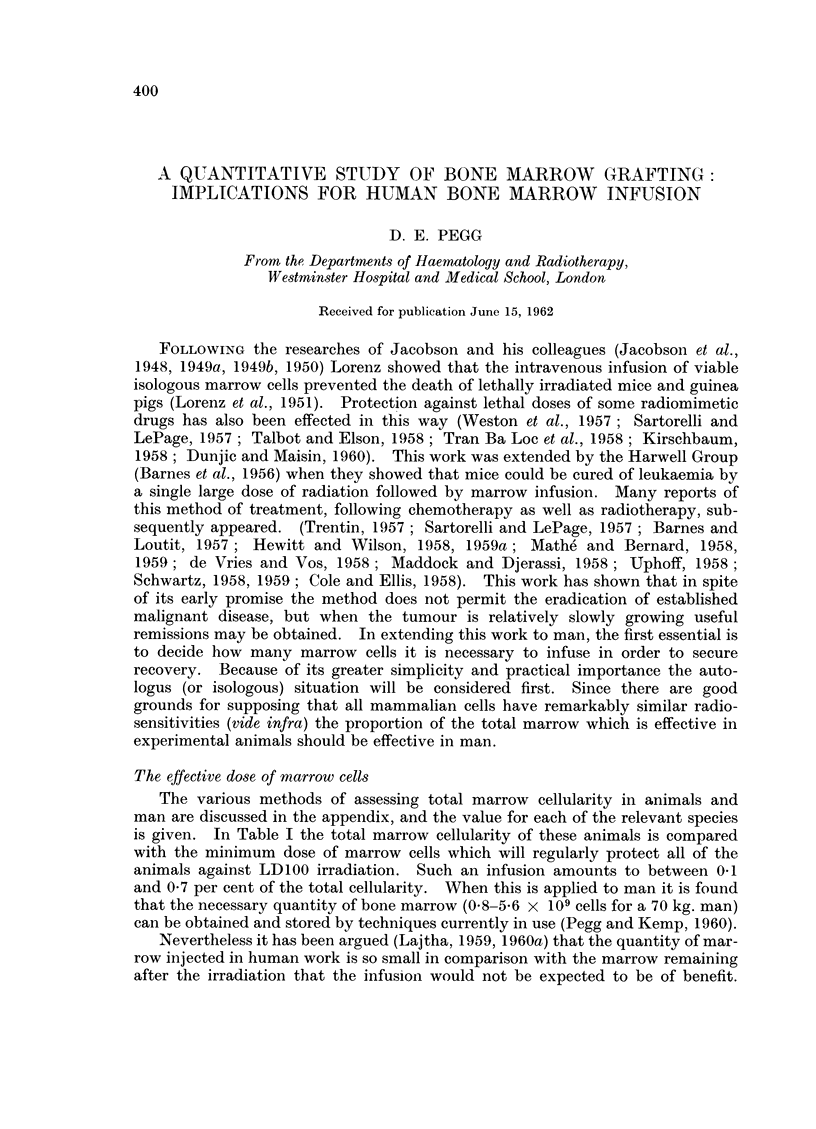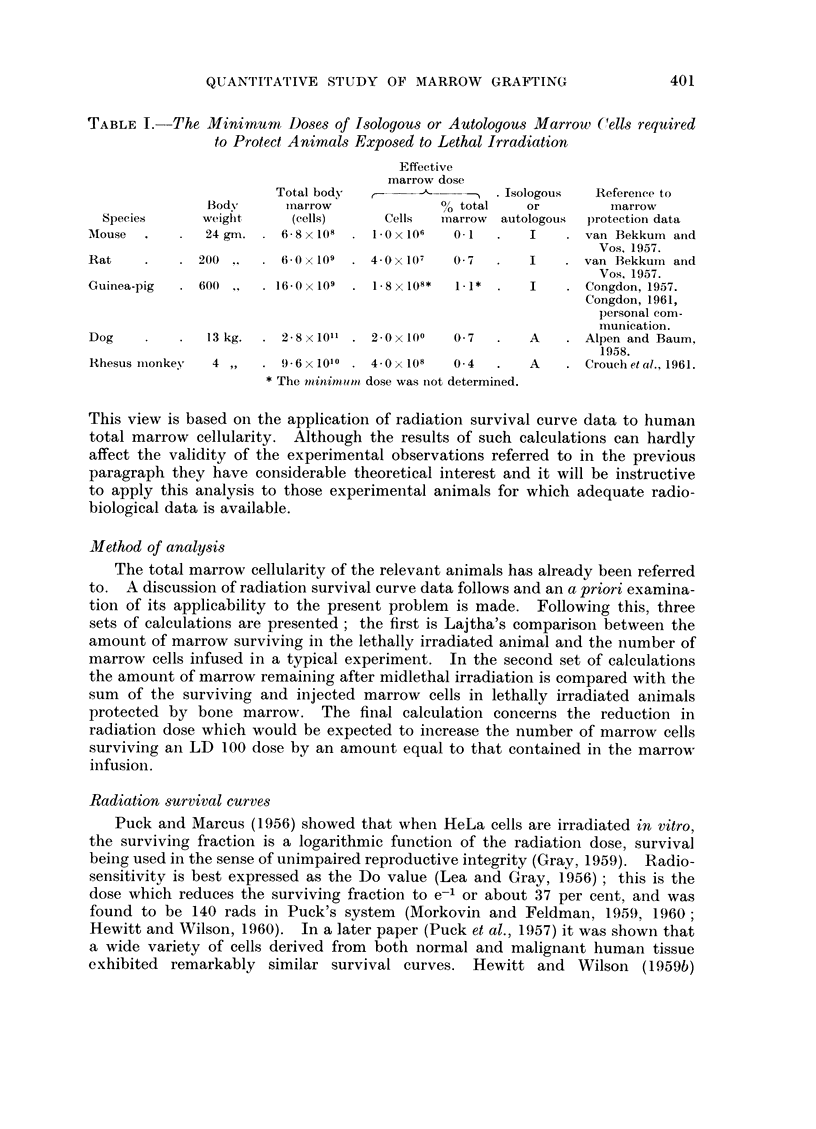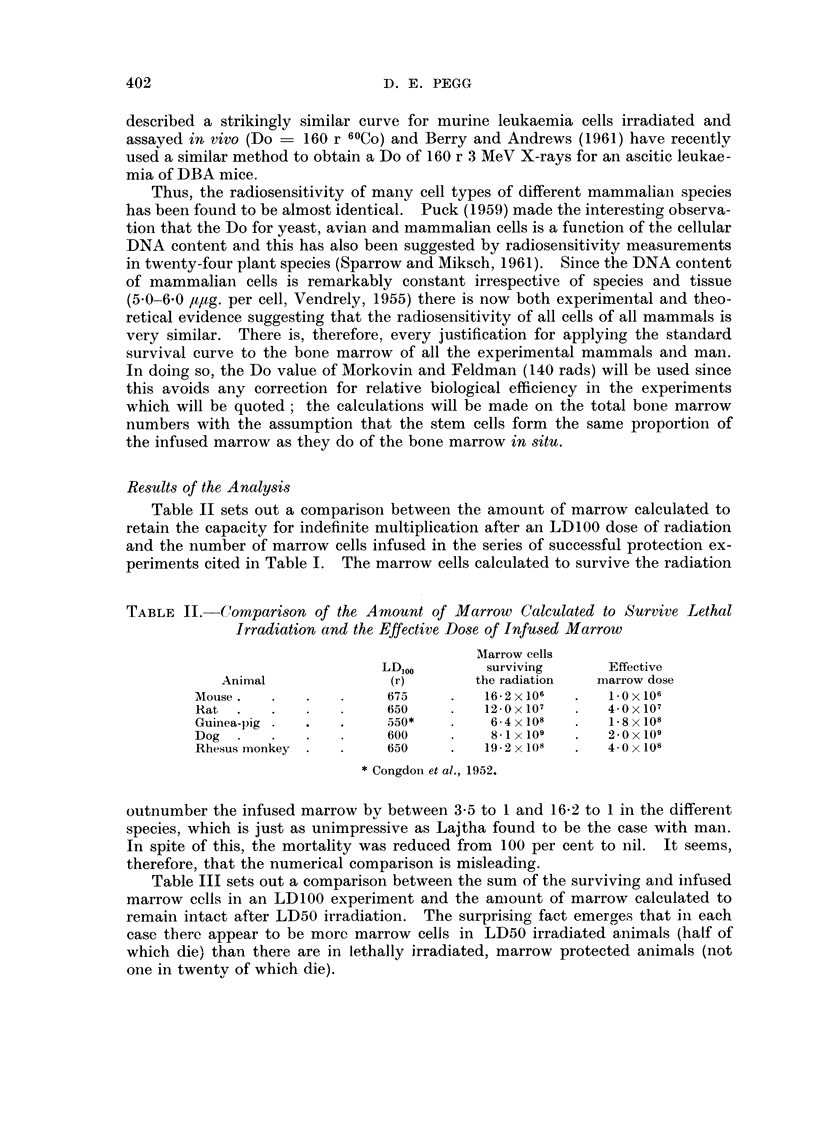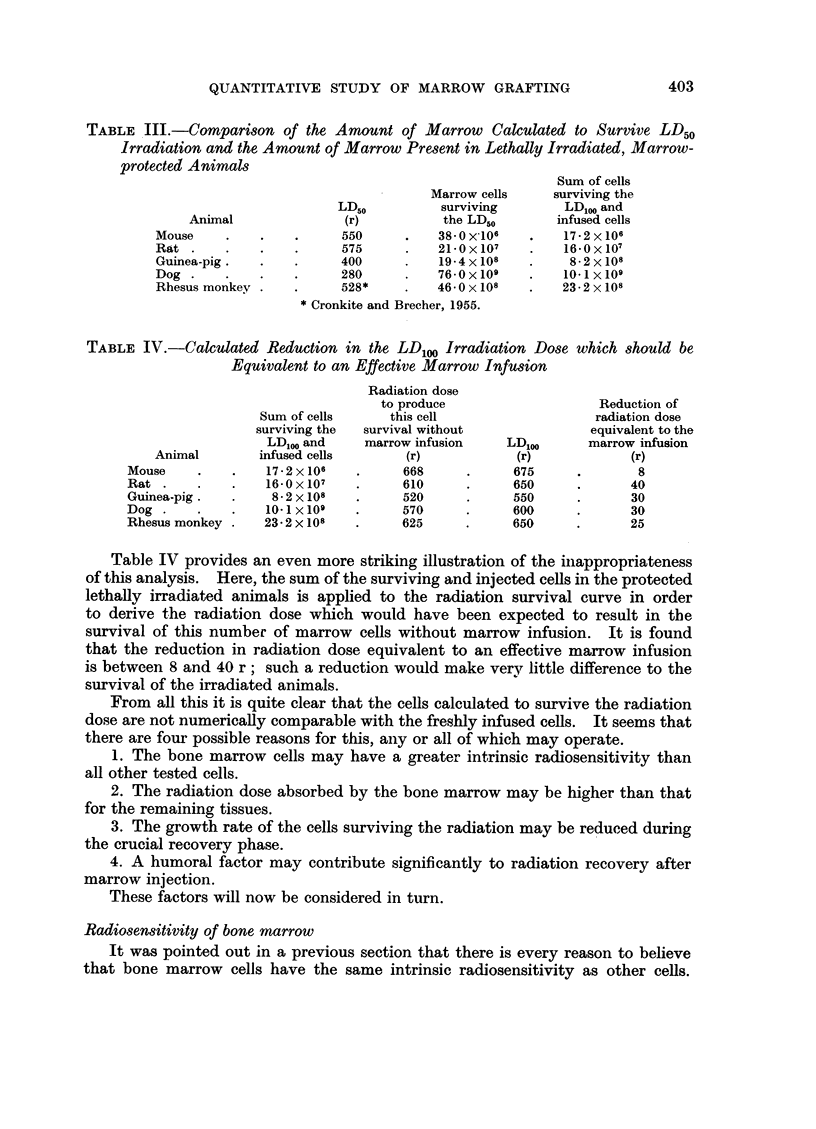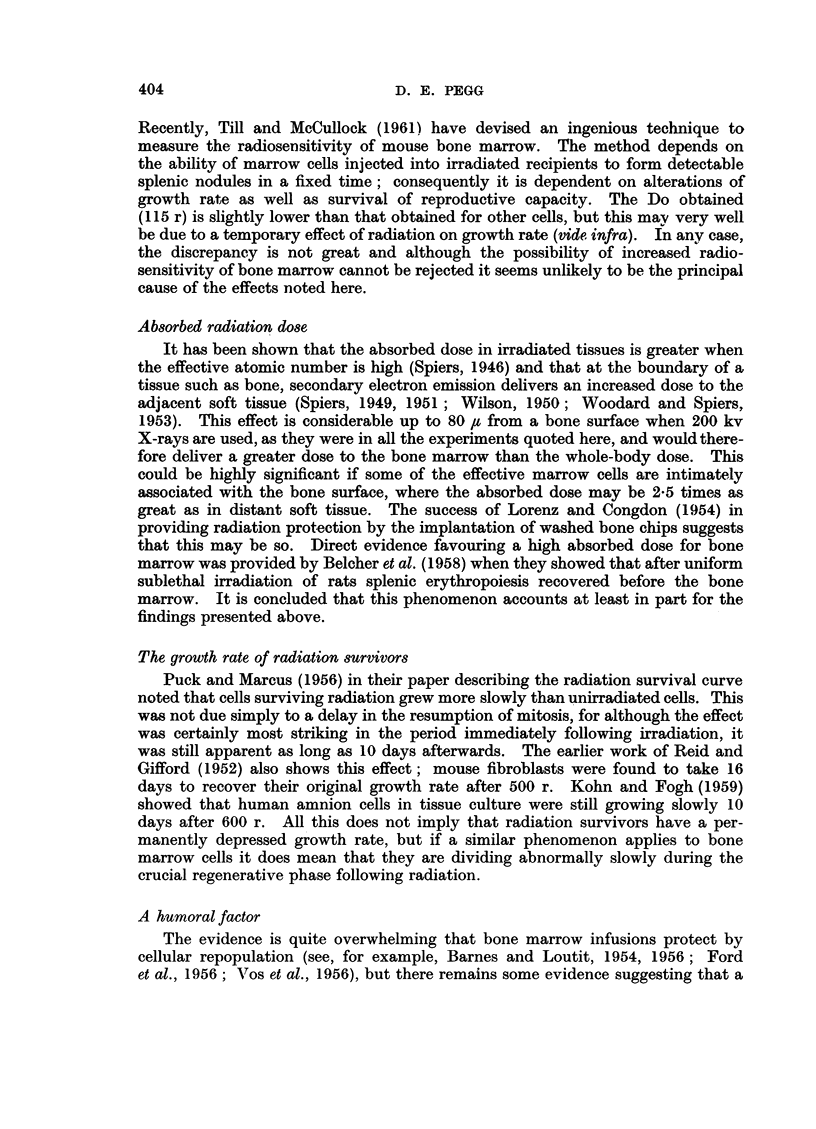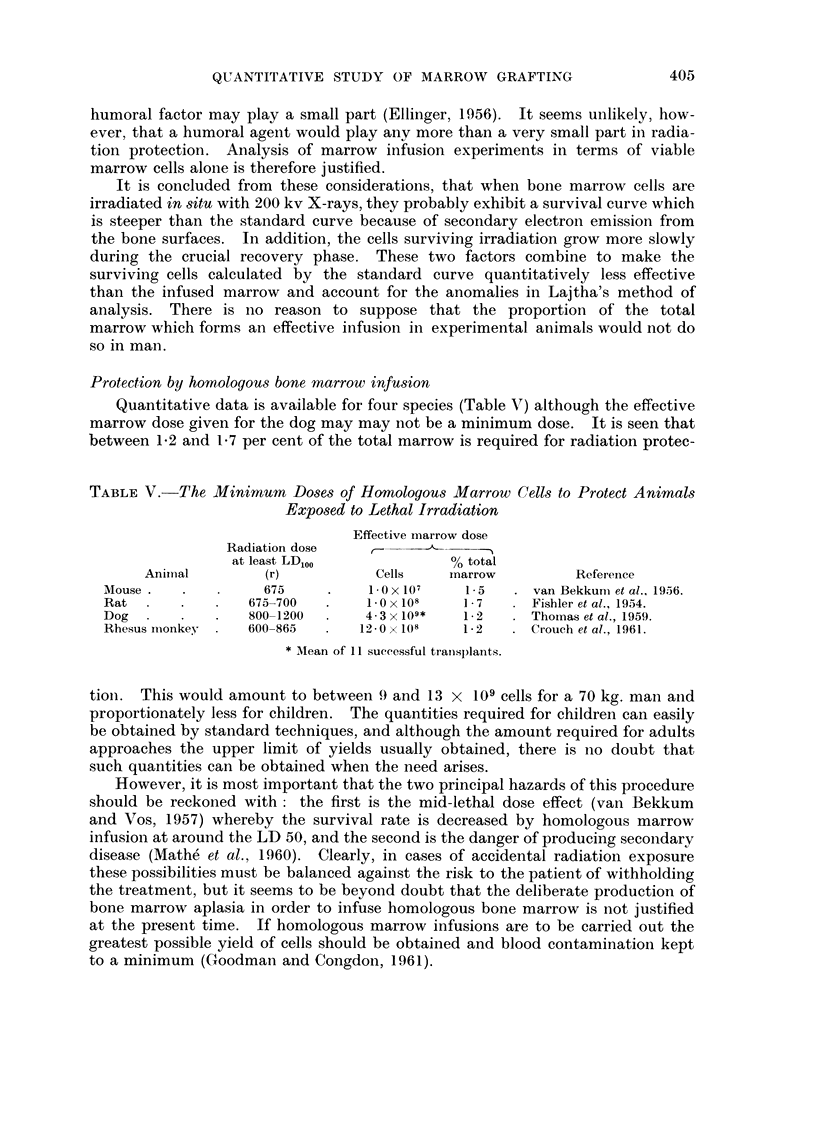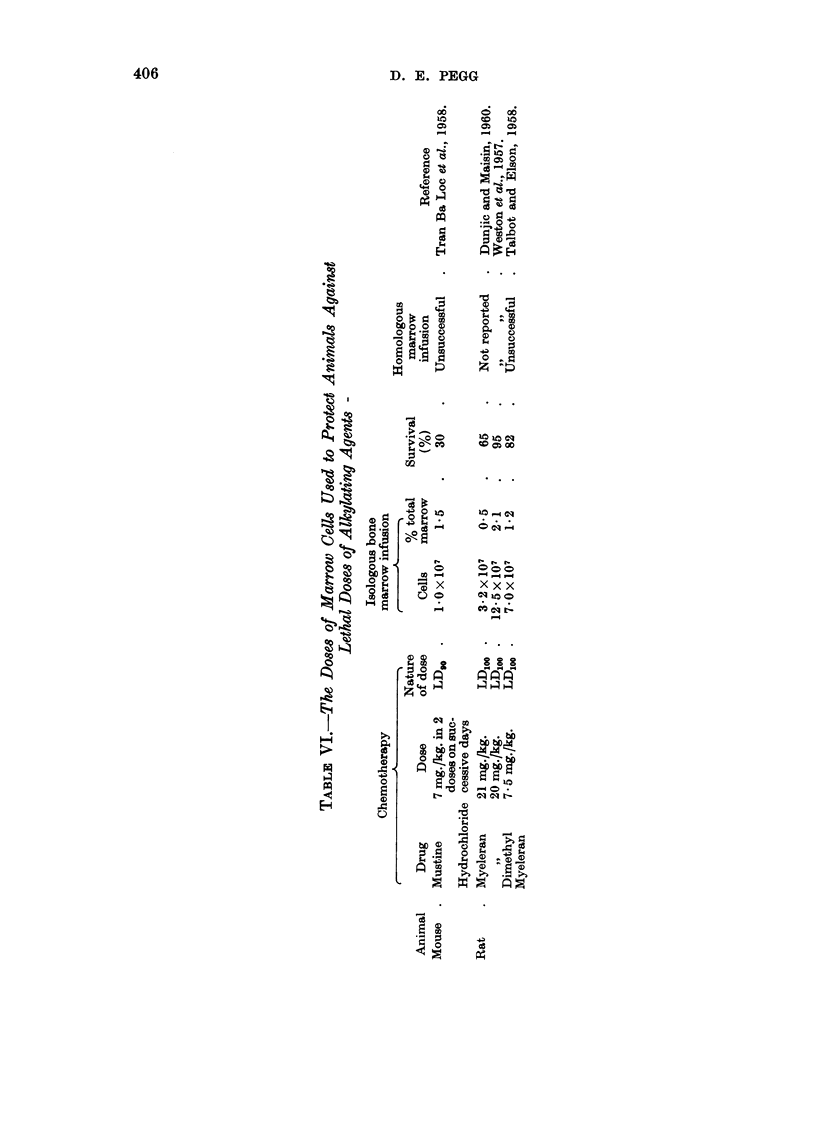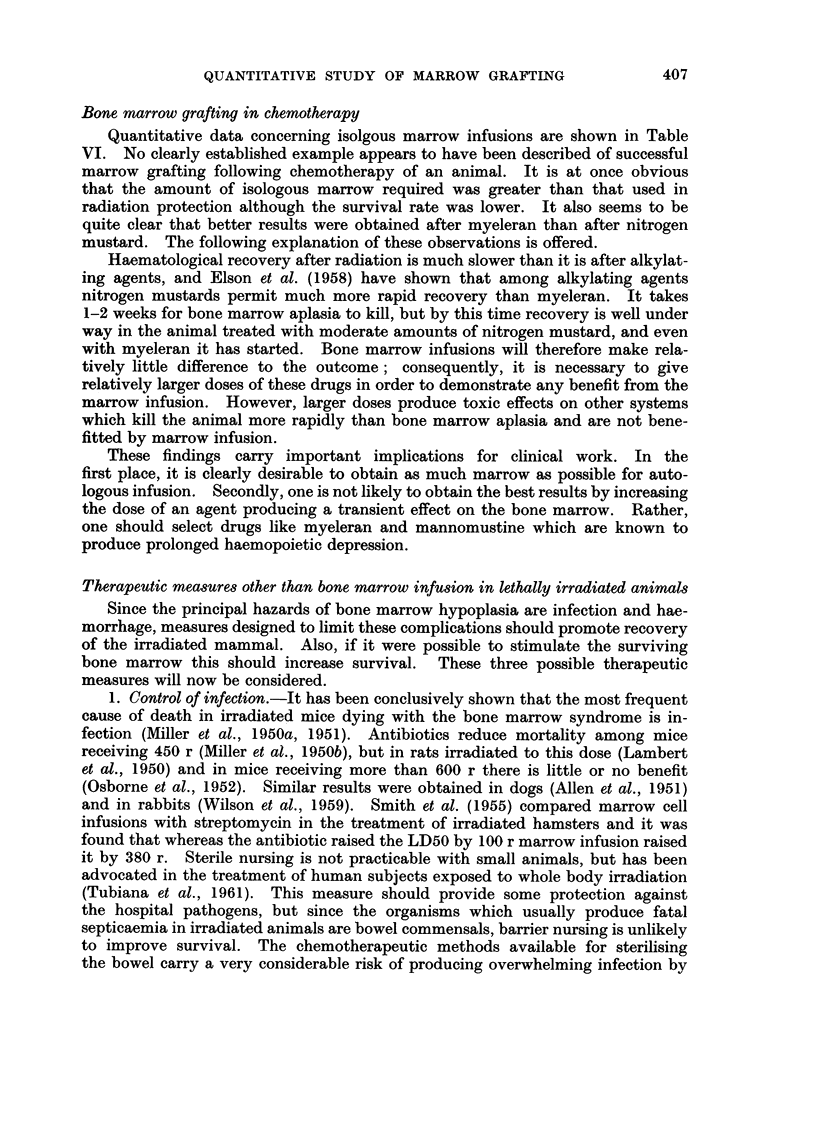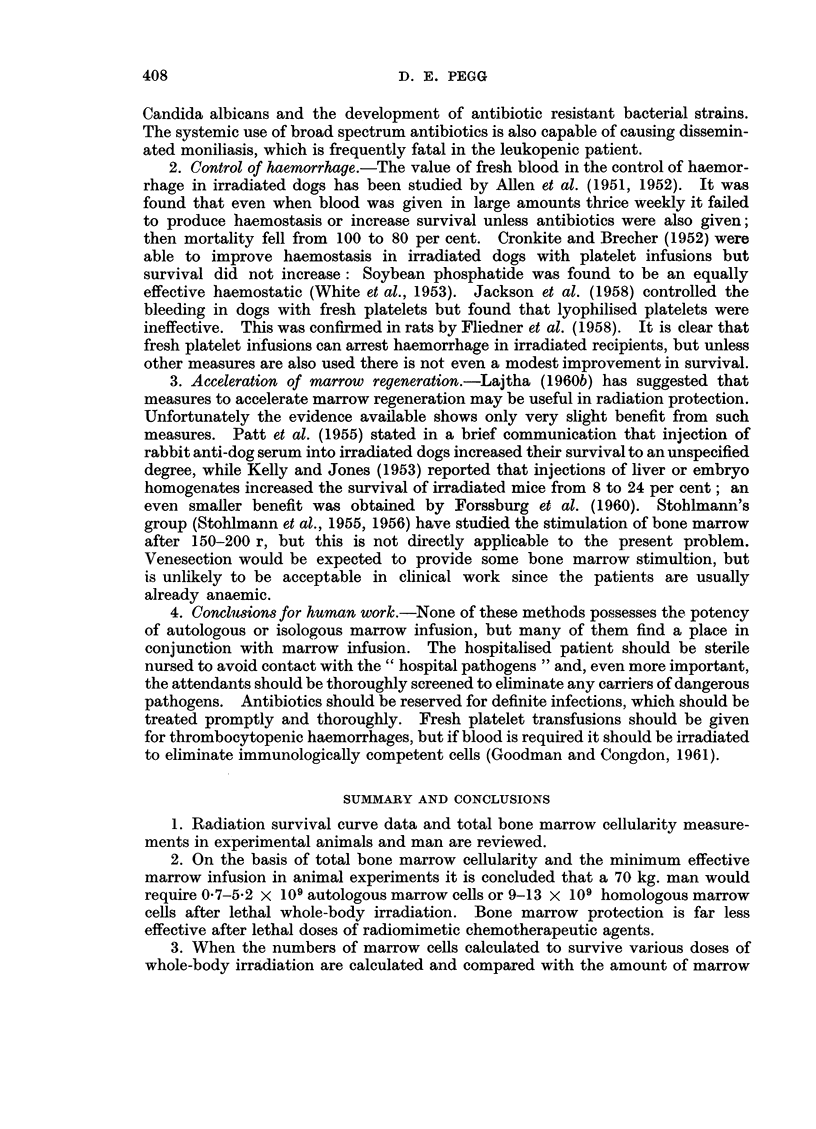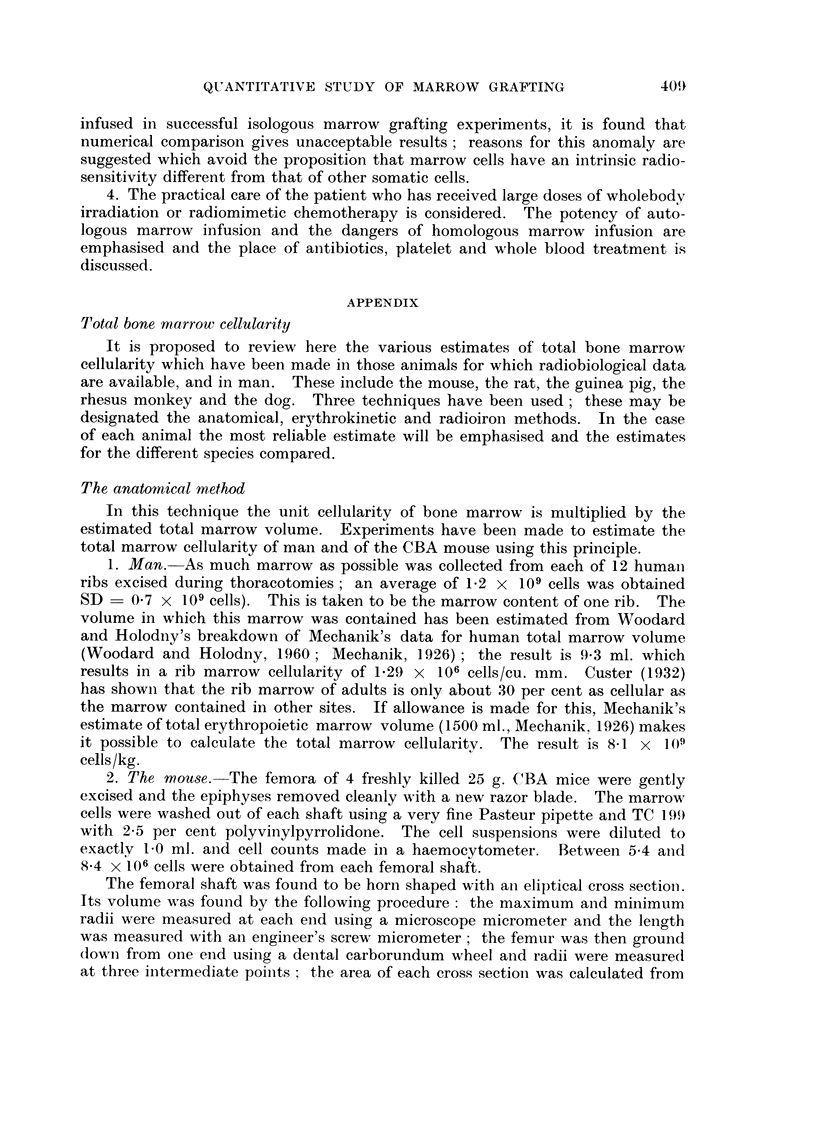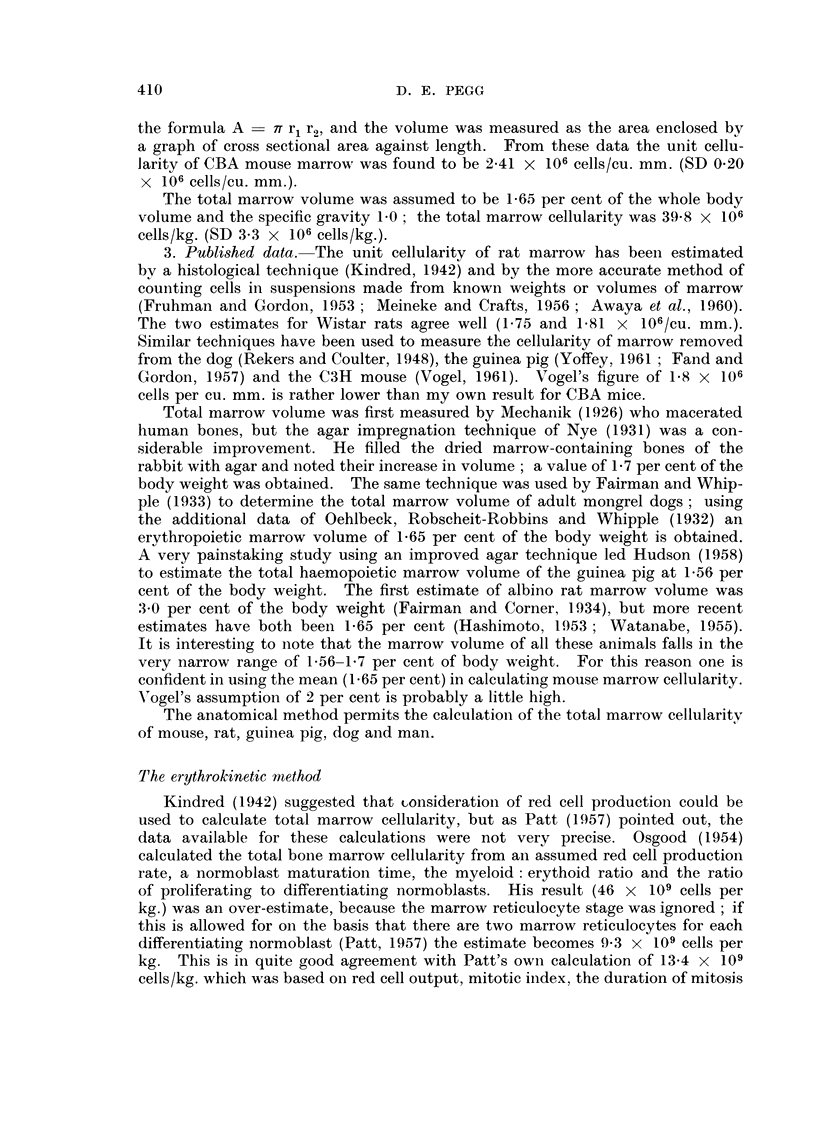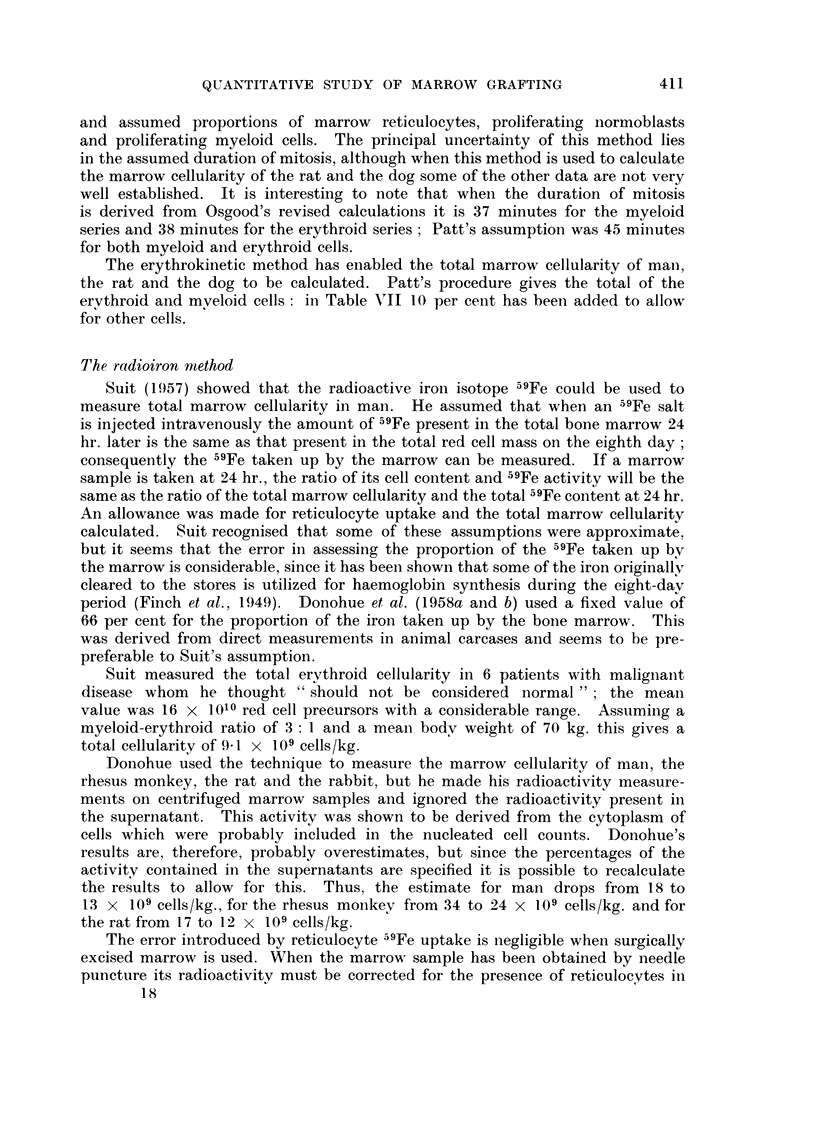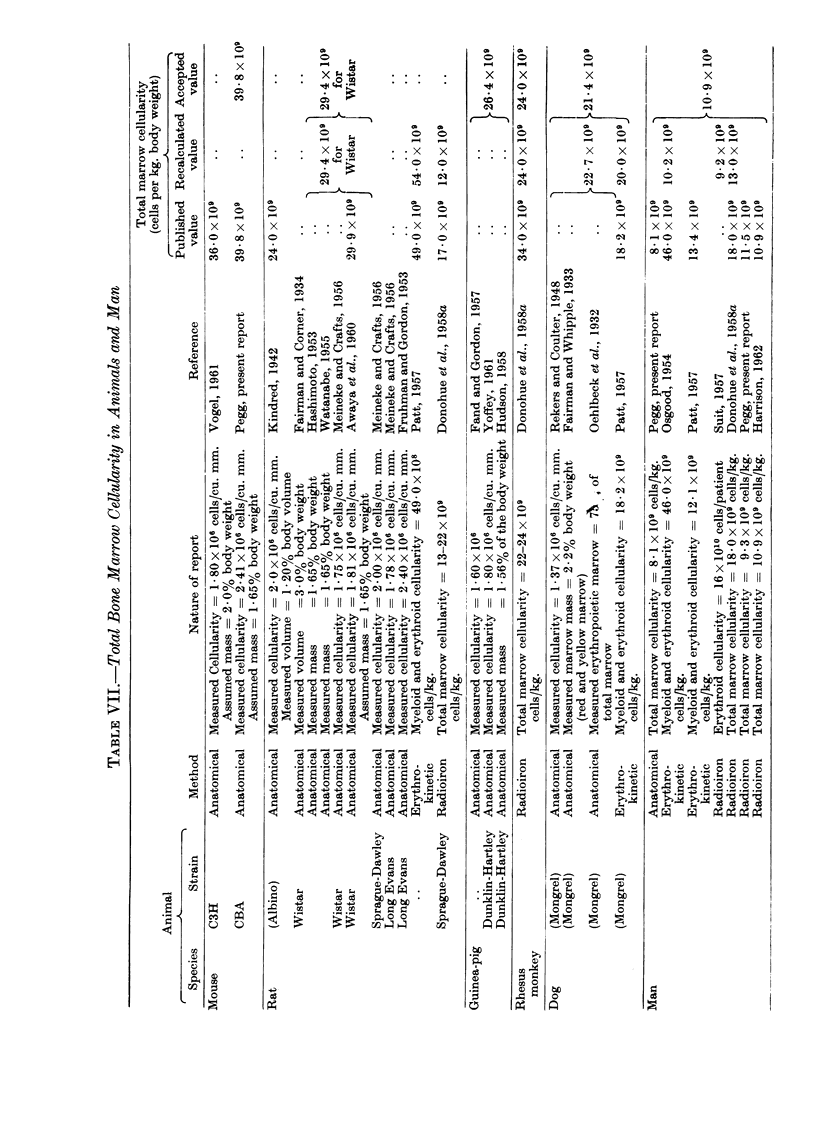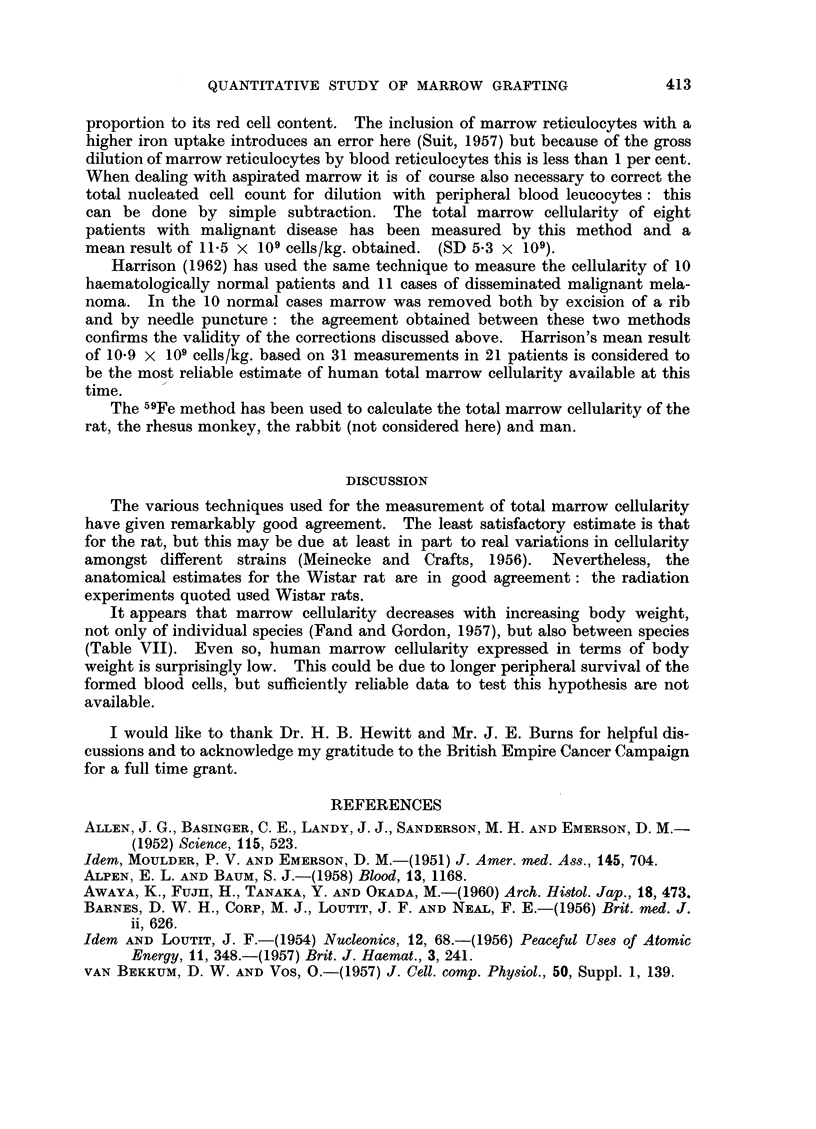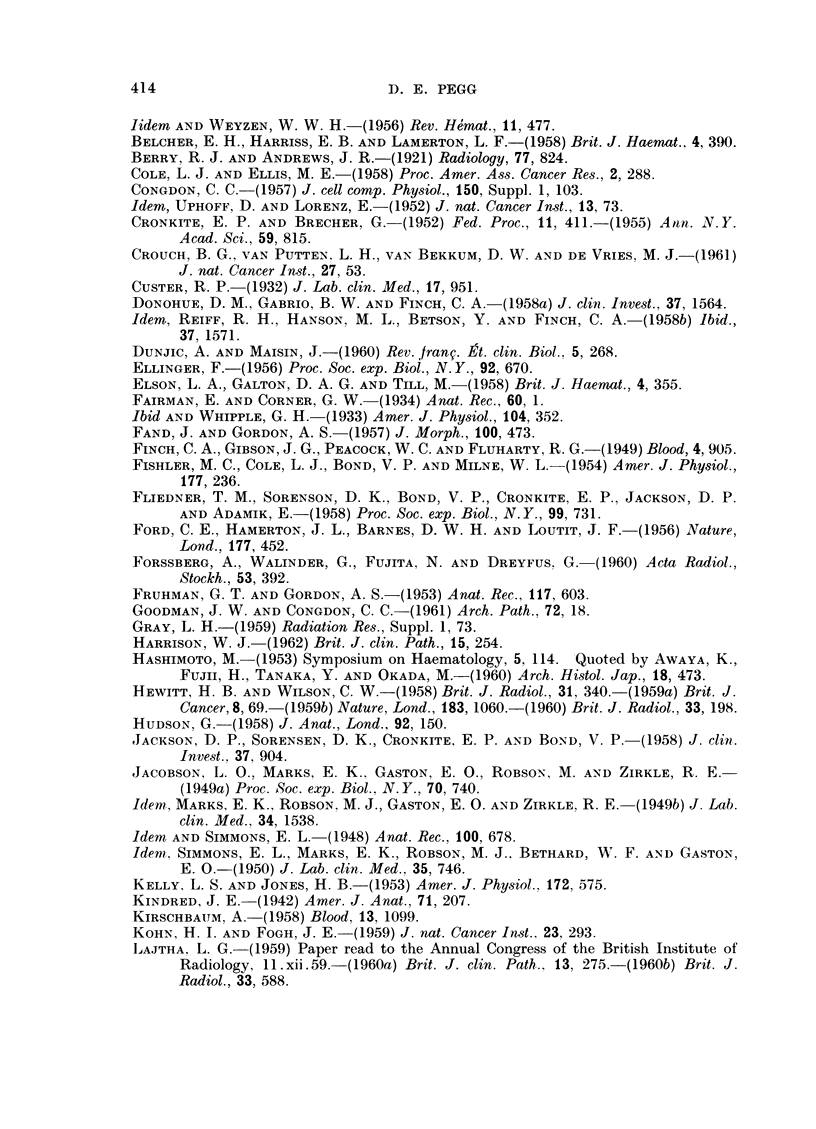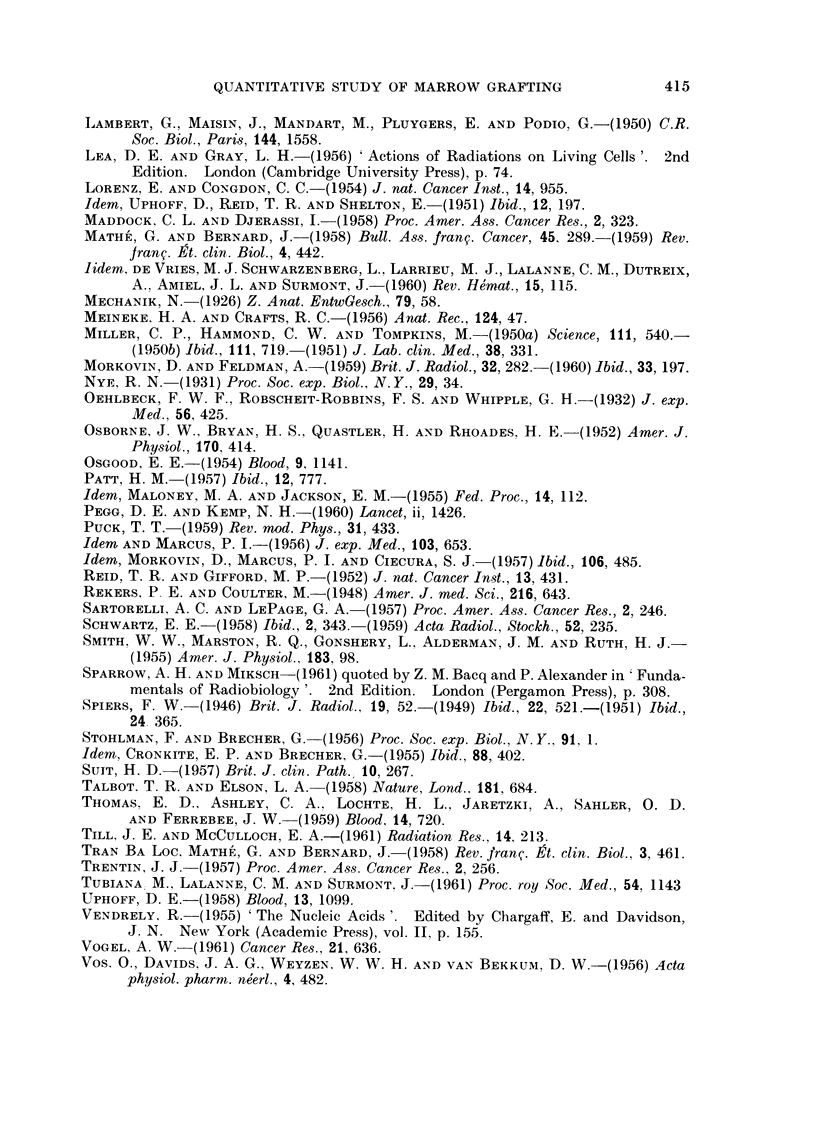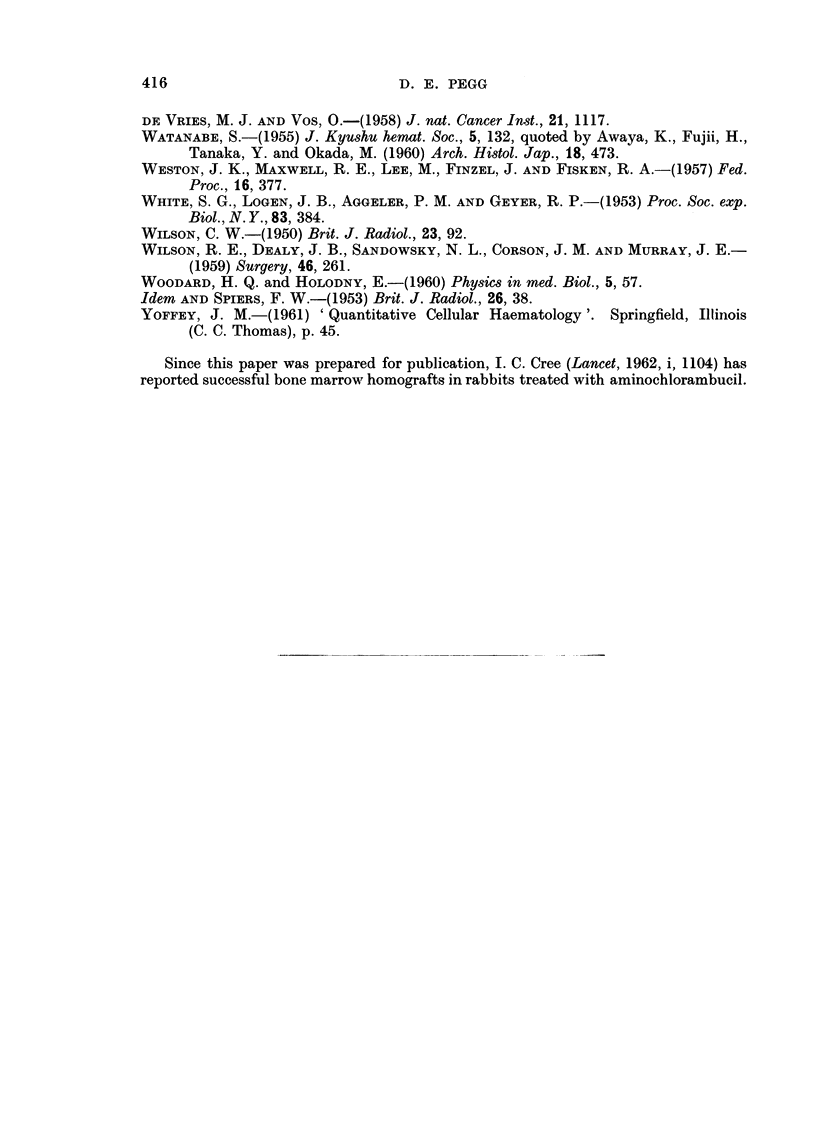# A Quantitative Study of Bone Marrow Grafting: Implications for Human Bone Marrow Infusion

**DOI:** 10.1038/bjc.1962.46

**Published:** 1962-09

**Authors:** D. E. Pegg


### A QUANTITATIVE STUDY OF BONE MARROW GRAFTING : IMPLICATIONS FOR HUMAN BONE MARROW INFUSION

D. E. PEGG

*From the Departments of Haematology and Radiotherapy, Westminster Hospital and Medical School, London*

Received for publication June 15, 1962

FOLLOWING the researches of Jacobson and his colleagues (Jacobson *et al.*, 1948, 1949*a*, 1949*b*, 1950) Lorenz showed that the intravenous infusion of viable isologous marrow cells prevented the death of lethally irradiated mice and guinea pigs (Lorenz *et al.*, 1951). Protection against lethal doses of some radiomimetic drugs has also been effected in this way (Weston *et al.*, 1957 ; Sartorelli and LePage, 1957 ; Talbot and Elson, 1958 ; Tran Ba Loc *et al.*, 1958 ; Kirschbaum, 1958 ; Dunjic and Maisin, 1960). This work was extended by the Harwell Group (Barnes *et al.*, 1956) when they showed that mice could be cured of leukaemia by a single large dose of radiation followed by marrow infusion. Many reports of this method of treatment, following chemotherapy as well as radiotherapy, subsequently appeared. (Trentin, 1957 ; Sartorelli and LePage, 1957 ; Barnes and Loutit, 1957 ; Hewitt and Wilson, 1958, 1959*a* ; Mathé and Bernard, 1958, 1959 ; de Vries and Vos, 1958 ; Maddock and Djerassi, 1958 ; Uphoff, 1958 ; Schwartz, 1958, 1959 ; Cole and Ellis, 1958). This work has shown that in spite of its early promise the method does not permit the eradication of established malignant disease, but when the tumour is relatively slowly growing useful remissions may be obtained. In extending this work to man, the first essential is to decide how many marrow cells it is necessary to infuse in order to secure recovery. Because of its greater simplicity and practical importance the autologus (or isologous) situation will be considered first. Since there are good grounds for supposing that all mammalian cells have remarkably similar radiosensitivities (*vide infra*) the proportion of the total marrow which is effective in experimental animals should be effective in man.

*The effective dose of marrow cells*

The various methods of assessing total marrow cellularity in animals and man are discussed in the appendix, and the value for each of the relevant species is given. In Table I the total marrow cellularity of these animals is compared with the minimum dose of marrow cells which will regularly protect all of the animals against LD100 irradiation. Such an infusion amounts to between 0·1 and 0·7 per cent of the total cellularity. When this is applied to man it is found that the necessary quantity of bone marrow (0·8–5·6 $\times$ $10^9$ cells for a 70 kg. man) can be obtained and stored by techniques currently in use (Pegg and Kemp, 1960).

Nevertheless it has been argued (Lajtha, 1959, 1960*a*) that the quantity of marrow injected in human work is so small in comparison with the marrow remaining after the irradiation that the infusion would not be expected to be of benefit.

TABLE I.—*The Minimum Doses of Isologous or Autologous Marrow Cells required to Protect Animals Exposed to Lethal Irradiation*

| Species | Body weight | Total body marrow (cells) | Effective marrow dose: Cells | Effective marrow dose: % total marrow | Isologous or autologous | Reference to marrow protection data |
|---|---|---|---|---|---|---|
| Mouse | 24 gm. | $6 \cdot 8 \times 10^8$ | $1 \cdot 0 \times 10^6$ | 0·1 | I | van Bekkum and Vos, 1957. |
| Rat | 200 ,, | $6 \cdot 0 \times 10^9$ | $4 \cdot 0 \times 10^7$ | 0·7 | I | van Bekkum and Vos, 1957. |
| Guinea-pig | 600 ,, | $16 \cdot 0 \times 10^9$ | $1 \cdot 8 \times 10^8$* | 1·1* | I | Congdon, 1957. Congdon, 1961, personal communication. |
| Dog | 13 kg. | $2 \cdot 8 \times 10^{11}$ | $2 \cdot 0 \times 10^{10}$ | 0·7 | A | Alpen and Baum, 1958. |
| Rhesus monkey | 4 ,, | $9 \cdot 6 \times 10^{10}$ | $4 \cdot 0 \times 10^8$ | 0·4 | A | Crouch *et al.*, 1961. |

* The *minimum* dose was not determined.

This view is based on the application of radiation survival curve data to human total marrow cellularity. Although the results of such calculations can hardly affect the validity of the experimental observations referred to in the previous paragraph they have considerable theoretical interest and it will be instructive to apply this analysis to those experimental animals for which adequate radiobiological data is available.

*Method of analysis*

The total marrow cellularity of the relevant animals has already been referred to. A discussion of radiation survival curve data follows and an *a priori* examination of its applicability to the present problem is made. Following this, three sets of calculations are presented ; the first is Lajtha's comparison between the amount of marrow surviving in the lethally irradiated animal and the number of marrow cells infused in a typical experiment. In the second set of calculations the amount of marrow remaining after midlethal irradiation is compared with the sum of the surviving and injected marrow cells in lethally irradiated animals protected by bone marrow. The final calculation concerns the reduction in radiation dose which would be expected to increase the number of marrow cells surviving an LD 100 dose by an amount equal to that contained in the marrow infusion.

*Radiation survival curves*

Puck and Marcus (1956) showed that when HeLa cells are irradiated *in vitro*, the surviving fraction is a logarithmic function of the radiation dose, survival being used in the sense of unimpaired reproductive integrity (Gray, 1959). Radiosensitivity is best expressed as the Do value (Lea and Gray, 1956) ; this is the dose which reduces the surviving fraction to $e^{-1}$ or about 37 per cent, and was found to be 140 rads in Puck's system (Morkovin and Feldman, 1959, 1960 ; Hewitt and Wilson, 1960). In a later paper (Puck *et al.*, 1957) it was shown that a wide variety of cells derived from both normal and malignant human tissue exhibited remarkably similar survival curves. Hewitt and Wilson (1959*b*)

described a strikingly similar curve for murine leukaemia cells irradiated and assayed *in vivo* (Do = 160 r $^{60}Co$) and Berry and Andrews (1961) have recently used a similar method to obtain a Do of 160 r 3 MeV X-rays for an ascitic leukaemia of DBA mice.

Thus, the radiosensitivity of many cell types of different mammalian species has been found to be almost identical. Puck (1959) made the interesting observation that the Do for yeast, avian and mammalian cells is a function of the cellular DNA content and this has also been suggested by radiosensitivity measurements in twenty-four plant species (Sparrow and Miksch, 1961). Since the DNA content of mammalian cells is remarkably constant irrespective of species and tissue (5·0–6·0 $\mu\mu$g. per cell, Vendrely, 1955) there is now both experimental and theoretical evidence suggesting that the radiosensitivity of all cells of all mammals is very similar. There is, therefore, every justification for applying the standard survival curve to the bone marrow of all the experimental mammals and man. In doing so, the Do value of Morkovin and Feldman (140 rads) will be used since this avoids any correction for relative biological efficiency in the experiments which will be quoted ; the calculations will be made on the total bone marrow numbers with the assumption that the stem cells form the same proportion of the infused marrow as they do of the bone marrow *in situ.*

*Results of the Analysis*

Table II sets out a comparison between the amount of marrow calculated to retain the capacity for indefinite multiplication after an LD100 dose of radiation and the number of marrow cells infused in the series of successful protection experiments cited in Table I. The marrow cells calculated to survive the radiation outnumber the infused marrow by between 3·5 to 1 and 16·2 to 1 in the different species, which is just as unimpressive as Lajtha found to be the case with man. In spite of this, the mortality was reduced from 100 per cent to nil. It seems, therefore, that the numerical comparison is misleading.

TABLE II.—*Comparison of the Amount of Marrow Calculated to Survive Lethal Irradiation and the Effective Dose of Infused Marrow*

| Animal | $LD_{100}$ (r) | Marrow cells surviving the radiation | Effective marrow dose |
|---|---|---|---|
| Mouse | 675 | $16 \cdot 2 \times 10^{6}$ | $1 \cdot 0 \times 10^{6}$ |
| Rat | 650 | $12 \cdot 0 \times 10^{7}$ | $4 \cdot 0 \times 10^{7}$ |
| Guinea-pig | 550* | $6 \cdot 4 \times 10^{8}$ | $1 \cdot 8 \times 10^{8}$ |
| Dog | 600 | $8 \cdot 1 \times 10^{9}$ | $2 \cdot 0 \times 10^{9}$ |
| Rhesus monkey | 650 | $19 \cdot 2 \times 10^{8}$ | $4 \cdot 0 \times 10^{8}$ |

* Congdon *et al.*, 1952.

Table III sets out a comparison between the sum of the surviving and infused marrow cells in an LD100 experiment and the amount of marrow calculated to remain intact after LD50 irradiation. The surprising fact emerges that in each case there appear to be more marrow cells in LD50 irradiated animals (half of which die) than there are in lethally irradiated, marrow protected animals (not one in twenty of which die).

TABLE III.—*Comparison of the Amount of Marrow Calculated to Survive $LD_{50}$ Irradiation and the Amount of Marrow Present in Lethally Irradiated, Marrow-protected Animals*

| Animal | $LD_{50}$ (r) | Marrow cells surviving the $LD_{50}$ | Sum of cells surviving the $LD_{100}$ and infused cells |
|---|---|---|---|
| Mouse | 550 | $38 \cdot 0 \times 10^6$ | $17 \cdot 2 \times 10^6$ |
| Rat | 575 | $21 \cdot 0 \times 10^7$ | $16 \cdot 0 \times 10^7$ |
| Guinea-pig | 400 | $19 \cdot 4 \times 10^8$ | $8 \cdot 2 \times 10^8$ |
| Dog | 280 | $76 \cdot 0 \times 10^9$ | $10 \cdot 1 \times 10^9$ |
| Rhesus monkey | 528* | $46 \cdot 0 \times 10^8$ | $23 \cdot 2 \times 10^8$ |

* Cronkite and Brecher, 1955.

TABLE IV.—*Calculated Reduction in the $LD_{100}$ Irradiation Dose which should be Equivalent to an Effective Marrow Infusion*

| Animal | Sum of cells surviving the $LD_{100}$ and infused cells | Radiation dose to produce this cell survival without marrow infusion (r) | $LD_{100}$ (r) | Reduction of radiation dose equivalent to the marrow infusion (r) |
|---|---|---|---|---|
| Mouse | $17 \cdot 2 \times 10^6$ | 668 | 675 | 8 |
| Rat | $16 \cdot 0 \times 10^7$ | 610 | 650 | 40 |
| Guinea-pig | $8 \cdot 2 \times 10^8$ | 520 | 550 | 30 |
| Dog | $10 \cdot 1 \times 10^9$ | 570 | 600 | 30 |
| Rhesus monkey | $23 \cdot 2 \times 10^8$ | 625 | 650 | 25 |

Table IV provides an even more striking illustration of the inappropriateness of this analysis. Here, the sum of the surviving and injected cells in the protected lethally irradiated animals is applied to the radiation survival curve in order to derive the radiation dose which would have been expected to result in the survival of this number of marrow cells without marrow infusion. It is found that the reduction in radiation dose equivalent to an effective marrow infusion is between 8 and 40 r ; such a reduction would make very little difference to the survival of the irradiated animals.

From all this it is quite clear that the cells calculated to survive the radiation dose are not numerically comparable with the freshly infused cells. It seems that there are four possible reasons for this, any or all of which may operate.

1. The bone marrow cells may have a greater intrinsic radiosensitivity than all other tested cells.
2. The radiation dose absorbed by the bone marrow may be higher than that for the remaining tissues.
3. The growth rate of the cells surviving the radiation may be reduced during the crucial recovery phase.
4. A humoral factor may contribute significantly to radiation recovery after marrow injection.

These factors will now be considered in turn.

*Radiosensitivity of bone marrow*

It was pointed out in a previous section that there is every reason to believe that bone marrow cells have the same intrinsic radiosensitivity as other cells.

Recently, Till and McCullock (1961) have devised an ingenious technique to measure the radiosensitivity of mouse bone marrow. The method depends on the ability of marrow cells injected into irradiated recipients to form detectable splenic nodules in a fixed time; consequently it is dependent on alterations of growth rate as well as survival of reproductive capacity. The Do obtained (115 r) is slightly lower than that obtained for other cells, but this may very well be due to a temporary effect of radiation on growth rate (*vide infra*). In any case, the discrepancy is not great and although the possibility of increased radiosensitivity of bone marrow cannot be rejected it seems unlikely to be the principal cause of the effects noted here.

*Absorbed radiation dose*

It has been shown that the absorbed dose in irradiated tissues is greater when the effective atomic number is high (Spiers, 1946) and that at the boundary of a tissue such as bone, secondary electron emission delivers an increased dose to the adjacent soft tissue (Spiers, 1949, 1951; Wilson, 1950; Woodard and Spiers, 1953). This effect is considerable up to 80 $\mu$ from a bone surface when 200 kv X-rays are used, as they were in all the experiments quoted here, and would therefore deliver a greater dose to the bone marrow than the whole-body dose. This could be highly significant if some of the effective marrow cells are intimately associated with the bone surface, where the absorbed dose may be 2·5 times as great as in distant soft tissue. The success of Lorenz and Congdon (1954) in providing radiation protection by the implantation of washed bone chips suggests that this may be so. Direct evidence favouring a high absorbed dose for bone marrow was provided by Belcher *et al.* (1958) when they showed that after uniform sublethal irradiation of rats splenic erythropoiesis recovered before the bone marrow. It is concluded that this phenomenon accounts at least in part for the findings presented above.

*The growth rate of radiation survivors*

Puck and Marcus (1956) in their paper describing the radiation survival curve noted that cells surviving radiation grew more slowly than unirradiated cells. This was not due simply to a delay in the resumption of mitosis, for although the effect was certainly most striking in the period immediately following irradiation, it was still apparent as long as 10 days afterwards. The earlier work of Reid and Gifford (1952) also shows this effect; mouse fibroblasts were found to take 16 days to recover their original growth rate after 500 r. Kohn and Fogh (1959) showed that human amnion cells in tissue culture were still growing slowly 10 days after 600 r. All this does not imply that radiation survivors have a permanently depressed growth rate, but if a similar phenomenon applies to bone marrow cells it does mean that they are dividing abnormally slowly during the crucial regenerative phase following radiation.

*A humoral factor*

The evidence is quite overwhelming that bone marrow infusions protect by cellular repopulation (see, for example, Barnes and Loutit, 1954, 1956; Ford *et al.*, 1956; Vos *et al.*, 1956), but there remains some evidence suggesting that a humoral factor may play a small part (Ellinger, 1956). It seems unlikely, however, that a humoral agent would play any more than a very small part in radiation protection. Analysis of marrow infusion experiments in terms of viable marrow cells alone is therefore justified.

It is concluded from these considerations, that when bone marrow cells are irradiated *in situ* with 200 kv X-rays, they probably exhibit a survival curve which is steeper than the standard curve because of secondary electron emission from the bone surfaces. In addition, the cells surviving irradiation grow more slowly during the crucial recovery phase. These two factors combine to make the surviving cells calculated by the standard curve quantitatively less effective than the infused marrow and account for the anomalies in Lajtha's method of analysis. There is no reason to suppose that the proportion of the total marrow which forms an effective infusion in experimental animals would not do so in man.

*Protection by homologous bone marrow infusion*

Quantitative data is available for four species (Table V) although the effective marrow dose given for the dog may may not be a minimum dose. It is seen that between 1·2 and 1·7 per cent of the total marrow is required for radiation protection. This would amount to between 9 and 13 $\times$ $10^9$ cells for a 70 kg. man and proportionately less for children. The quantities required for children can easily be obtained by standard techniques, and although the amount required for adults approaches the upper limit of yields usually obtained, there is no doubt that such quantities can be obtained when the need arises.

TABLE V.—*The Minimum Doses of Homologous Marrow Cells to Protect Animals Exposed to Lethal Irradiation*

| Animal | Radiation dose at least $LD_{100}$ (r) | Effective marrow dose: Cells | Effective marrow dose: % total marrow | Reference |
|---|---|---|---|---|
| Mouse | 675 | $1 \cdot 0 \times 10^7$ | 1·5 | van Bekkum *et al.*, 1956. |
| Rat | 675–700 | $1 \cdot 0 \times 10^8$ | 1·7 | Fishler *et al.*, 1954. |
| Dog | 800–1200 | $4 \cdot 3 \times 10^9$* | 1·2 | Thomas *et al.*, 1959. |
| Rhesus monkey | 600–865 | $12 \cdot 0 \times 10^8$ | 1·2 | Crouch *et al.*, 1961. |

* Mean of 11 successful transplants.

However, it is most important that the two principal hazards of this procedure should be reckoned with : the first is the mid-lethal dose effect (van Bekkum and Vos, 1957) whereby the survival rate is decreased by homologous marrow infusion at around the LD 50, and the second is the danger of producing secondary disease (Mathé *et al.*, 1960). Clearly, in cases of accidental radiation exposure these possibilities must be balanced against the risk to the patient of withholding the treatment, but it seems to be beyond doubt that the deliberate production of bone marrow aplasia in order to infuse homologous bone marrow is not justified at the present time. If homologous marrow infusions are to be carried out the greatest possible yield of cells should be obtained and blood contamination kept to a minimum (Goodman and Congdon, 1961).

TABLE VI.—*The Doses of Marrow Cells Used to Protect Animals Against Lethal Doses of Alkylating Agents* -

| Animal | Chemotherapy | | | Isologous bone marrow infusion | | Survival (%) | Homologous marrow infusion | Reference |
|---|---|---|---|---|---|---|---|---|
| | Drug | Dose | Nature of dose | Cells | % total marrow | | | |
| Mouse | Mustine Hydrochloride | 7 mg./kg. in 2 doses on successive days | $LD_{90}$ | $1\cdot0\times10^7$ | 1·5 | 30 | Unsuccessful | Tran Ba Loc *et al.*, 1958. |
| Rat | Myeleran | 21 mg./kg. | $LD_{100}$ | $3\cdot2\times10^7$ | 0·5 | 65 | Not reported | Dunjic and Maisin, 1960. |
| | ,, | 20 mg./kg. | $LD_{100}$ | $12\cdot5\times10^7$ | 2·1 | 95 | ,, ,, | Weston *et al.*, 1957. |
| | Dimethyl Myeleran | 7·5 mg./kg. | $LD_{100}$ | $7\cdot0\times10^7$ | 1·2 | 82 | Unsuccessful | Talbot and Elson, 1958. |

*Bone marrow grafting in chemotherapy*

Quantitative data concerning isolgous marrow infusions are shown in Table VI. No clearly established example appears to have been described of successful marrow grafting following chemotherapy of an animal. It is at once obvious that the amount of isologous marrow required was greater than that used in radiation protection although the survival rate was lower. It also seems to be quite clear that better results were obtained after myeleran than after nitrogen mustard. The following explanation of these observations is offered.

Haematological recovery after radiation is much slower than it is after alkylating agents, and Elson *et al.* (1958) have shown that among alkylating agents nitrogen mustards permit much more rapid recovery than myeleran. It takes 1–2 weeks for bone marrow aplasia to kill, but by this time recovery is well under way in the animal treated with moderate amounts of nitrogen mustard, and even with myeleran it has started. Bone marrow infusions will therefore make relatively little difference to the outcome ; consequently, it is necessary to give relatively larger doses of these drugs in order to demonstrate any benefit from the marrow infusion. However, larger doses produce toxic effects on other systems which kill the animal more rapidly than bone marrow aplasia and are not benefitted by marrow infusion.

These findings carry important implications for clinical work. In the first place, it is clearly desirable to obtain as much marrow as possible for autologous infusion. Secondly, one is not likely to obtain the best results by increasing the dose of an agent producing a transient effect on the bone marrow. Rather, one should select drugs like myeleran and mannomustine which are known to produce prolonged haemopoietic depression.

*Therapeutic measures other than bone marrow infusion in lethally irradiated animals*

Since the principal hazards of bone marrow hypoplasia are infection and haemorrhage, measures designed to limit these complications should promote recovery of the irradiated mammal. Also, if it were possible to stimulate the surviving bone marrow this should increase survival. These three possible therapeutic measures will now be considered.

1. *Control of infection.*—It has been conclusively shown that the most frequent cause of death in irradiated mice dying with the bone marrow syndrome is infection (Miller *et al.*, 1950*a*, 1951). Antibiotics reduce mortality among mice receiving 450 r (Miller *et al.*, 1950*b*), but in rats irradiated to this dose (Lambert *et al.*, 1950) and in mice receiving more than 600 r there is little or no benefit (Osborne *et al.*, 1952). Similar results were obtained in dogs (Allen *et al.*, 1951) and in rabbits (Wilson *et al.*, 1959). Smith *et al.* (1955) compared marrow cell infusions with streptomycin in the treatment of irradiated hamsters and it was found that whereas the antibiotic raised the LD50 by 100 r marrow infusion raised it by 380 r. Sterile nursing is not practicable with small animals, but has been advocated in the treatment of human subjects exposed to whole body irradiation (Tubiana *et al.*, 1961). This measure should provide some protection against the hospital pathogens, but since the organisms which usually produce fatal septicaemia in irradiated animals are bowel commensals, barrier nursing is unlikely to improve survival. The chemotherapeutic methods available for sterilising the bowel carry a very considerable risk of producing overwhelming infection by

Candida albicans and the development of antibiotic resistant bacterial strains. The systemic use of broad spectrum antibiotics is also capable of causing disseminated moniliasis, which is frequently fatal in the leukopenic patient.

2. *Control of haemorrhage*.—The value of fresh blood in the control of haemorrhage in irradiated dogs has been studied by Allen *et al.* (1951, 1952). It was found that even when blood was given in large amounts thrice weekly it failed to produce haemostasis or increase survival unless antibiotics were also given; then mortality fell from 100 to 80 per cent. Cronkite and Brecher (1952) were able to improve haemostasis in irradiated dogs with platelet infusions but survival did not increase: Soybean phosphatide was found to be an equally effective haemostatic (White *et al.*, 1953). Jackson *et al.* (1958) controlled the bleeding in dogs with fresh platelets but found that lyophilised platelets were ineffective. This was confirmed in rats by Fliedner *et al.* (1958). It is clear that fresh platelet infusions can arrest haemorrhage in irradiated recipients, but unless other measures are also used there is not even a modest improvement in survival.

3. *Acceleration of marrow regeneration*.—Lajtha (1960*b*) has suggested that measures to accelerate marrow regeneration may be useful in radiation protection. Unfortunately the evidence available shows only very slight benefit from such measures. Patt *et al.* (1955) stated in a brief communication that injection of rabbit anti-dog serum into irradiated dogs increased their survival to an unspecified degree, while Kelly and Jones (1953) reported that injections of liver or embryo homogenates increased the survival of irradiated mice from 8 to 24 per cent; an even smaller benefit was obtained by Forssburg *et al.* (1960). Stohlmann's group (Stohlmann *et al.*, 1955, 1956) have studied the stimulation of bone marrow after 150–200 r, but this is not directly applicable to the present problem. Venesection would be expected to provide some bone marrow stimultion, but is unlikely to be acceptable in clinical work since the patients are usually already anaemic.

4. *Conclusions for human work*.—None of these methods possesses the potency of autologous or isologous marrow infusion, but many of them find a place in conjunction with marrow infusion. The hospitalised patient should be sterile nursed to avoid contact with the " hospital pathogens " and, even more important, the attendants should be thoroughly screened to eliminate any carriers of dangerous pathogens. Antibiotics should be reserved for definite infections, which should be treated promptly and thoroughly. Fresh platelet transfusions should be given for thrombocytopenic haemorrhages, but if blood is required it should be irradiated to eliminate immunologically competent cells (Goodman and Congdon, 1961).

### SUMMARY AND CONCLUSIONS

1. Radiation survival curve data and total bone marrow cellularity measurements in experimental animals and man are reviewed.

2. On the basis of total bone marrow cellularity and the minimum effective marrow infusion in animal experiments it is concluded that a 70 kg. man would require $0{\cdot}7–5{\cdot}2 \times 10^9$ autologous marrow cells or $9–13 \times 10^9$ homologous marrow cells after lethal whole-body irradiation. Bone marrow protection is far less effective after lethal doses of radiomimetic chemotherapeutic agents.

3. When the numbers of marrow cells calculated to survive various doses of whole-body irradiation are calculated and compared with the amount of marrow infused in successful isologous marrow grafting experiments, it is found that numerical comparison gives unacceptable results ; reasons for this anomaly are suggested which avoid the proposition that marrow cells have an intrinsic radiosensitivity different from that of other somatic cells.

4. The practical care of the patient who has received large doses of wholebody irradiation or radiomimetic chemotherapy is considered. The potency of autologous marrow infusion and the dangers of homologous marrow infusion are emphasised and the place of antibiotics, platelet and whole blood treatment is discussed.

### APPENDIX

*Total bone marrow cellularity*

It is proposed to review here the various estimates of total bone marrow cellularity which have been made in those animals for which radiobiological data are available, and in man. These include the mouse, the rat, the guinea pig, the rhesus monkey and the dog. Three techniques have been used ; these may be designated the anatomical, erythrokinetic and radioiron methods. In the case of each animal the most reliable estimate will be emphasised and the estimates for the different species compared.

*The anatomical method*

In this technique the unit cellularity of bone marrow is multiplied by the estimated total marrow volume. Experiments have been made to estimate the total marrow cellularity of man and of the CBA mouse using this principle.

1. *Man.*—As much marrow as possible was collected from each of 12 human ribs excised during thoracotomies ; an average of $1\cdot2 \times 10^9$ cells was obtained SD = $0\cdot7 \times 10^9$ cells). This is taken to be the marrow content of one rib. The volume in which this marrow was contained has been estimated from Woodard and Holodny's breakdown of Mechanik's data for human total marrow volume (Woodard and Holodny, 1960 ; Mechanik, 1926) ; the result is 9·3 ml. which results in a rib marrow cellularity of $1\cdot29 \times 10^6$ cells/cu. mm. Custer (1932) has shown that the rib marrow of adults is only about 30 per cent as cellular as the marrow contained in other sites. If allowance is made for this, Mechanik's estimate of total erythropoietic marrow volume (1500 ml., Mechanik, 1926) makes it possible to calculate the total marrow cellularity. The result is $8\cdot1 \times 10^9$ cells/kg.

2. *The mouse.*—The femora of 4 freshly killed 25 g. CBA mice were gently excised and the epiphyses removed cleanly with a new razor blade. The marrow cells were washed out of each shaft using a very fine Pasteur pipette and TC 199 with 2·5 per cent polyvinylpyrrolidone. The cell suspensions were diluted to exactly 1·0 ml. and cell counts made in a haemocytometer. Between 5·4 and $8\cdot4 \times 10^6$ cells were obtained from each femoral shaft.

The femoral shaft was found to be horn shaped with an eliptical cross section. Its volume was found by the following procedure : the maximum and minimum radii were measured at each end using a microscope micrometer and the length was measured with an engineer's screw micrometer ; the femur was then ground down from one end using a dental carborundum wheel and radii were measured at three intermediate points ; the area of each cross section was calculated from the formula $A = \pi r_1 r_2$, and the volume was measured as the area enclosed by a graph of cross sectional area against length. From these data the unit cellularity of CBA mouse marrow was found to be $2{\cdot}41 \times 10^6$ cells/cu. mm. (SD $0{\cdot}20 \times 10^6$ cells/cu. mm.).

The total marrow volume was assumed to be 1·65 per cent of the whole body volume and the specific gravity 1·0 ; the total marrow cellularity was $39{\cdot}8 \times 10^6$ cells/kg. (SD $3{\cdot}3 \times 10^6$ cells/kg.).

3. *Published data.*—The unit cellularity of rat marrow has been estimated by a histological technique (Kindred, 1942) and by the more accurate method of counting cells in suspensions made from known weights or volumes of marrow (Fruhman and Gordon, 1953 ; Meineke and Crafts, 1956 ; Awaya *et al.*, 1960). The two estimates for Wistar rats agree well (1·75 and $1{\cdot}81 \times 10^6$/cu. mm.). Similar techniques have been used to measure the cellularity of marrow removed from the dog (Rekers and Coulter, 1948), the guinea pig (Yoffey, 1961 ; Fand and Gordon, 1957) and the C3H mouse (Vogel, 1961). Vogel's figure of $1{\cdot}8 \times 10^6$ cells per cu. mm. is rather lower than my own result for CBA mice.

Total marrow volume was first measured by Mechanik (1926) who macerated human bones, but the agar impregnation technique of Nye (1931) was a considerable improvement. He filled the dried marrow-containing bones of the rabbit with agar and noted their increase in volume ; a value of 1·7 per cent of the body weight was obtained. The same technique was used by Fairman and Whipple (1933) to determine the total marrow volume of adult mongrel dogs ; using the additional data of Oehlbeck, Robscheit-Robbins and Whipple (1932) an erythropoietic marrow volume of 1·65 per cent of the body weight is obtained. A very painstaking study using an improved agar technique led Hudson (1958) to estimate the total haemopoietic marrow volume of the guinea pig at 1·56 per cent of the body weight. The first estimate of albino rat marrow volume was 3·0 per cent of the body weight (Fairman and Corner, 1934), but more recent estimates have both been 1·65 per cent (Hashimoto, 1953 ; Watanabe, 1955). It is interesting to note that the marrow volume of all these animals falls in the very narrow range of 1·56–1·7 per cent of body weight. For this reason one is confident in using the mean (1·65 per cent) in calculating mouse marrow cellularity. Vogel's assumption of 2 per cent is probably a little high.

The anatomical method permits the calculation of the total marrow cellularity of mouse, rat, guinea pig, dog and man.

*The erythrokinetic method*

Kindred (1942) suggested that consideration of red cell production could be used to calculate total marrow cellularity, but as Patt (1957) pointed out, the data available for these calculations were not very precise. Osgood (1954) calculated the total bone marrow cellularity from an assumed red cell production rate, a normoblast maturation time, the myeloid : erythoid ratio and the ratio of proliferating to differentiating normoblasts. His result ($46 \times 10^9$ cells per kg.) was an over-estimate, because the marrow reticulocyte stage was ignored ; if this is allowed for on the basis that there are two marrow reticulocytes for each differentiating normoblast (Patt, 1957) the estimate becomes $9{\cdot}3 \times 10^9$ cells per kg. This is in quite good agreement with Patt's own calculation of $13{\cdot}4 \times 10^9$ cells/kg. which was based on red cell output, mitotic index, the duration of mitosis and assumed proportions of marrow reticulocytes, proliferating normoblasts and proliferating myeloid cells. The principal uncertainty of this method lies in the assumed duration of mitosis, although when this method is used to calculate the marrow cellularity of the rat and the dog some of the other data are not very well established. It is interesting to note that when the duration of mitosis is derived from Osgood's revised calculations it is 37 minutes for the myeloid series and 38 minutes for the erythroid series ; Patt's assumption was 45 minutes for both myeloid and erythroid cells.

The erythrokinetic method has enabled the total marrow cellularity of man, the rat and the dog to be calculated. Patt's procedure gives the total of the erythroid and myeloid cells : in Table VII 10 per cent has been added to allow for other cells.

*The radioiron method*

Suit (1957) showed that the radioactive iron isotope $^{59}Fe$ could be used to measure total marrow cellularity in man. He assumed that when an $^{59}Fe$ salt is injected intravenously the amount of $^{59}Fe$ present in the total bone marrow 24 hr. later is the same as that present in the total red cell mass on the eighth day ; consequently the $^{59}Fe$ taken up by the marrow can be measured. If a marrow sample is taken at 24 hr., the ratio of its cell content and $^{59}Fe$ activity will be the same as the ratio of the total marrow cellularity and the total $^{59}Fe$ content at 24 hr. An allowance was made for reticulocyte uptake and the total marrow cellularity calculated. Suit recognised that some of these assumptions were approximate, but it seems that the error in assessing the proportion of the $^{59}Fe$ taken up by the marrow is considerable, since it has been shown that some of the iron originally cleared to the stores is utilized for haemoglobin synthesis during the eight-day period (Finch *et al.*, 1949). Donohue *et al.* (1958*a* and *b*) used a fixed value of 66 per cent for the proportion of the iron taken up by the bone marrow. This was derived from direct measurements in animal carcases and seems to be prepreferable to Suit's assumption.

Suit measured the total erythroid cellularity in 6 patients with malignant disease whom he thought " should not be considered normal " ; the mean value was $16 \times 10^{10}$ red cell precursors with a considerable range. Assuming a myeloid-erythroid ratio of 3 : 1 and a mean body weight of 70 kg. this gives a total cellularity of $9{\cdot}1 \times 10^9$ cells/kg.

Donohue used the technique to measure the marrow cellularity of man, the rhesus monkey, the rat and the rabbit, but he made his radioactivity measurements on centrifuged marrow samples and ignored the radioactivity present in the supernatant. This activity was shown to be derived from the cytoplasm of cells which were probably included in the nucleated cell counts. Donohue's results are, therefore, probably overestimates, but since the percentages of the activity contained in the supernatants are specified it is possible to recalculate the results to allow for this. Thus, the estimate for man drops from 18 to $13 \times 10^9$ cells/kg., for the rhesus monkey from 34 to $24 \times 10^9$ cells/kg. and for the rat from 17 to $12 \times 10^9$ cells/kg.

The error introduced by reticulocyte $^{59}Fe$ uptake is negligible when surgically excised marrow is used. When the marrow sample has been obtained by needle puncture its radioactivity must be corrected for the presence of reticulocytes in

### TABLE VII.—*Total Bone Marrow Cellularity in Animals and Man*

| Animal: Species | Animal: Strain | Method | Nature of report | Reference | Total marrow cellularity (cells per kg. body weight): Published value | Recalculated value | Accepted value |
|---|---|---|---|---|---|---|---|
| Mouse | C3H | Anatomical | Measured Cellularity = $1\cdot80\times10^{6}$ cells/cu. mm. Assumed mass = $2\cdot0\%$ body weight | Vogel, 1961 | $36\cdot0\times10^{9}$ | .. | .. |
| | CBA | Anatomical | Measured cellularity = $2\cdot41\times10^{6}$ cells/cu. mm. Assumed mass = $1\cdot65\%$ body weight | Pegg, present report | $39\cdot8\times10^{9}$ | .. | $39\cdot8\times10^{9}$ |
| Rat | (Albino) | Anatomical | Measured cellularity = $2\cdot0\times10^{6}$ cells/cu. mm. Measured volume = $1\cdot20\%$ body volume | Kindred, 1942 | $24\cdot0\times10^{9}$ | .. | .. |
| | Wistar | Anatomical | Measured volume = $3\cdot0\%$ body weight | Fairman and Corner, 1934 | .. | .. | .. |
| | | Anatomical | Measured mass = $1\cdot65\%$ body weight | Hashimoto, 1953 | .. | | |
| | | Anatomical | Measured mass = $1\cdot65\%$ body weight | Watanabe, 1955 | .. | | |
| | Wistar | Anatomical | Measured cellularity = $1\cdot75\times10^{6}$ cells/cu. mm. | Meineke and Crafts, 1956 | .. | $29\cdot4\times10^{9}$ for Wistar | $29\cdot4\times10^{9}$ for Wistar |
| | Wistar | Anatomical | Measured cellularity = $1\cdot81\times10^{6}$ cells/cu. mm. Assumed mass = $1\cdot65\%$ body weight | Awaya *et al.*, 1960 | $29\cdot9\times10^{9}$ | | |
| | Sprague-Dawley | Anatomical | Measured cellularity = $2\cdot00\times10^{6}$ cells/cu. mm. | Meineke and Crafts, 1956 | | | |
| | Long Evans | Anatomical | Measured cellularity = $1\cdot78\times10^{6}$ cells/cu. mm. | Meineke and Crafts, 1956 | .. | .. | .. |
| | Long Evans | Anatomical | Measured cellularity = $2\cdot40\times10^{6}$ cells/cu. mm. | Fruhman and Gordon, 1953 | .. | .. | .. |
| | .. | Erythro-kinetic | Myeloid and erythroid cellularity = $49\cdot0\times10^{8}$ cells/kg. | Patt, 1957 | $49\cdot0\times10^{9}$ | $54\cdot0\times10^{9}$ | .. |
| | Sprague-Dawley | Radioiron | Total marrow cellularity = 13–22 $\times10^{9}$ cells/kg. | Donohue *et al.*, 1958*a* | $17\cdot0\times10^{9}$ | $12\cdot0\times10^{9}$ | .. |
| Guinea-pig | .. | Anatomical | Measured cellularity = $1\cdot60\times10^{6}$ | Fand and Gordon, 1957 | .. | .. | |
| | Dunklin-Hartley | Anatomical | Measured cellularity = $1\cdot80\times10^{6}$ cells/cu. mm. | Yoffey, 1961 | .. | .. | $26\cdot4\times10^{9}$ |
| | Dunklin-Hartley | Anatomical | Measured mass = $1\cdot56\%$ of the body weight | Hudson, 1958 | .. | .. | |
| Rhesus monkey | | Radioiron | Total marrow cellularity = 22–24 $\times10^{9}$ cells/kg. | Donohue *et al.*, 1958*a* | $34\cdot0\times10^{9}$ | $24\cdot0\times10^{9}$ | $24\cdot0\times10^{9}$ |
| Dog | (Mongrel) | Anatomical | Measured cellularity = $1\cdot37\times10^{6}$ cells/cu. mm. | Rekers and Coulter, 1948 | .. | | |
| | (Mongrel) | Anatomical | Measured marrow mass = $2\cdot2\%$ body weight (red and yellow marrow) | Fairman and Whipple, 1933 | .. | $22\cdot7\times10^{9}$ | $21\cdot4\times10^{9}$ |
| | (Mongrel) | Anatomical | Measured erythropoietic marrow = 75 , of total marrow | Oehlbeck *et al.*, 1932 | .. | | |
| | (Mongrel) | Erythro-kinetic | Myeloid and erythroid cellularity = $18\cdot2\times10^{9}$ cells/kg. | Patt, 1957 | $18\cdot2\times10^{9}$ | $20\cdot0\times10^{9}$ | |
| Man | | Anatomical | Total marrow cellularity = $8\cdot1\times10^{9}$ cells/kg. | Pegg, present report | $8\cdot1\times10^{9}$ | | |
| | | Erythro-kinetic | Myeloid and erythroid cellularity = $46\cdot0\times10^{9}$ cells/kg. | Osgood, 1954 | $46\cdot0\times10^{9}$ | $10\cdot2\times10^{9}$ | |
| | | Erythro-kinetic | Myeloid and erythroid cellularity = $12\cdot1\times10^{9}$ cells/kg. | Patt, 1957 | $13\cdot4\times10^{9}$ | | |
| | | Radioiron | Erythroid cellularity = $16\times10^{10}$ cells/patient | Suit, 1957 | .. | $9\cdot2\times10^{9}$ | $10\cdot9\times10^{9}$ |
| | | Radioiron | Total marrow cellularity = $18\cdot0\times10^{9}$ cells/kg. | Donohue *et al.*, 1958*a* | $18\cdot0\times10^{9}$ | $13\cdot0\times10^{9}$ | |
| | | Radioiron | Total marrow cellularity = $9\cdot3\times10^{9}$ cells/kg. | Pegg, present report | $11\cdot5\times10^{9}$ | | |
| | | Radioiron | Total marrow cellularity = $10\cdot9\times10^{9}$ cells/kg. | Harrison, 1962 | $10\cdot9\times10^{9}$ | | |

proportion to its red cell content. The inclusion of marrow reticulocytes with a higher iron uptake introduces an error here (Suit, 1957) but because of the gross dilution of marrow reticulocytes by blood reticulocytes this is less than 1 per cent. When dealing with aspirated marrow it is of course also necessary to correct the total nucleated cell count for dilution with peripheral blood leucocytes : this can be done by simple subtraction. The total marrow cellularity of eight patients with malignant disease has been measured by this method and a mean result of $11{\cdot}5 \times 10^9$ cells/kg. obtained. (SD $5{\cdot}3 \times 10^9$).

Harrison (1962) has used the same technique to measure the cellularity of 10 haematologically normal patients and 11 cases of disseminated malignant melanoma. In the 10 normal cases marrow was removed both by excision of a rib and by needle puncture : the agreement obtained between these two methods confirms the validity of the corrections discussed above. Harrison's mean result of $10{\cdot}9 \times 10^9$ cells/kg. based on 31 measurements in 21 patients is considered to be the most reliable estimate of human total marrow cellularity available at this time.

The $^{59}Fe$ method has been used to calculate the total marrow cellularity of the rat, the rhesus monkey, the rabbit (not considered here) and man.

### DISCUSSION

The various techniques used for the measurement of total marrow cellularity have given remarkably good agreement. The least satisfactory estimate is that for the rat, but this may be due at least in part to real variations in cellularity amongst different strains (Meinecke and Crafts, 1956). Nevertheless, the anatomical estimates for the Wistar rat are in good agreement : the radiation experiments quoted used Wistar rats.

It appears that marrow cellularity decreases with increasing body weight, not only of individual species (Fand and Gordon, 1957), but also between species (Table VII). Even so, human marrow cellularity expressed in terms of body weight is surprisingly low. This could be due to longer peripheral survival of the formed blood cells, but sufficiently reliable data to test this hypothesis are not available.

I would like to thank Dr. H. B. Hewitt and Mr. J. E. Burns for helpful discussions and to acknowledge my gratitude to the British Empire Cancer Campaign for a full time grant.

### REFERENCES

ALLEN, J. G., BASINGER, C. E., LANDY, J. J., SANDERSON, M. H. AND EMERSON, D. M.—(1952) *Science*, **115**, 523.
*Idem*, MOULDER, P. V. AND EMERSON, D. M.—(1951) *J. Amer. med. Ass.*, **145**, 704.
ALPEN, E. L. AND BAUM, S. J.—(1958) *Blood*, **13**, 1168.
AWAYA, K., FUJII, H., TANAKA, Y. AND OKADA, M.—(1960) *Arch. Histol. Jap.*, **18**, 473.
BARNES, D. W. H., CORP, M. J., LOUTIT, J. F. AND NEAL, F. E.—(1956) *Brit. med. J.* ii, 626.
*Idem* AND LOUTIT, J. F.—(1954) *Nucleonics*, **12**, 68.—(1956) *Peaceful Uses of Atomic Energy*, **11**, 348.—(1957) *Brit. J. Haemat.*, **3**, 241.
VAN BEKKUM, D. W. AND VOS, O.—(1957) *J. Cell. comp. Physiol.*, **50**, Suppl. 1, 139.

*Iidem* AND WEYZEN, W. W. H.—(1956) *Rev. Hémat.*, **11**, 477.
BELCHER, E. H., HARRISS, E. B. AND LAMERTON, L. F.—(1958) *Brit. J. Haemat.*, **4**, 390.
BERRY, R. J. AND ANDREWS, J. R.—(1921) *Radiology*, **77**, 824.
COLE, L. J. AND ELLIS, M. E.—(1958) *Proc. Amer. Ass. Cancer Res.*, **2**, 288.
CONGDON, C. C.—(1957) *J. cell comp. Physiol.*, **150**, Suppl. 1, 103.
*Idem*, UPHOFF, D. AND LORENZ, E.—(1952) *J. nat. Cancer Inst.*, **13**, 73.
CRONKITE, E. P. AND BRECHER, G.—(1952) *Fed. Proc.*, **11**, 411.—(1955) *Ann. N.Y. Acad. Sci.*, **59**, 815.
CROUCH, B. G., VAN PUTTEN, L. H., VAN BEKKUM, D. W. AND DE VRIES, M. J.—(1961) *J. nat. Cancer Inst.*, **27**, 53.
CUSTER, R. P.—(1932) *J. Lab. clin. Med.*, **17**, 951.
DONOHUE, D. M., GABRIO, B. W. AND FINCH, C. A.—(1958*a*) *J. clin. Invest.*, **37**, 1564.
*Idem*, REIFF, R. H., HANSON, M. L., BETSON, Y. AND FINCH, C. A.—(1958*b*) *Ibid.*, **37**, 1571.
DUNJIC, A. AND MAISIN, J.—(1960) *Rev. franç. Ét. clin. Biol.*, **5**, 268.
ELLINGER, F.—(1956) *Proc. Soc. exp. Biol., N.Y.*, **92**, 670.
ELSON, L. A., GALTON, D. A. G. AND TILL, M.—(1958) *Brit. J. Haemat.*, **4**, 355.
FAIRMAN, E. AND CORNER, G. W.—(1934) *Anat. Rec.*, **60**, 1.
*Ibid* AND WHIPPLE, G. H.—(1933) *Amer. J. Physiol.*, **104**, 352.
FAND, J. AND GORDON, A. S.—(1957) *J. Morph.*, **100**, 473.
FINCH, C. A., GIBSON, J. G., PEACOCK, W. C. AND FLUHARTY, R. G.—(1949) *Blood*, **4**, 905.
FISHLER, M. C., COLE, L. J., BOND, V. P. AND MILNE, W. L.—(1954) *Amer. J. Physiol.*, **177**, 236.
FLIEDNER, T. M., SORENSON, D. K., BOND, V. P., CRONKITE, E. P., JACKSON, D. P. AND ADAMIK, E.—(1958) *Proc. Soc. exp. Biol., N.Y.*, **99**, 731.
FORD, C. E., HAMERTON, J. L., BARNES, D. W. H. AND LOUTIT, J. F.—(1956) *Nature, Lond.*, **177**, 452.
FORSSBERG, A., WALINDER, G., FUJITA, N. AND DREYFUS, G.—(1960) *Acta Radiol., Stockh.*, **53**, 392.
FRUHMAN, G. T. AND GORDON, A. S.—(1953) *Anat. Rec.*, **117**, 603.
GOODMAN, J. W. AND CONGDON, C. C.—(1961) *Arch. Path.*, **72**, 18.
GRAY, L. H.—(1959) *Radiation Res.*, Suppl. 1, 73.
HARRISON, W. J.—(1962) *Brit. J. clin. Path.*, **15**, 254.
HASHIMOTO, M.—(1953) Symposium on Haematology, **5**, 114. Quoted by AWAYA, K., FUJII, H., TANAKA, Y. AND OKADA, M.—(1960) *Arch. Histol. Jap.*, **18**, 473.
HEWITT, H. B. AND WILSON, C. W.—(1958) *Brit. J. Radiol.*, **31**, 340.—(1959*a*) *Brit. J. Cancer*, **8**, 69.—(1959*b*) *Nature, Lond.*, **183**, 1060.—(1960) *Brit. J. Radiol.*, **33**, 198.
HUDSON, G.—(1958) *J. Anat., Lond.*, **92**, 150.
JACKSON, D. P., SORENSEN, D. K., CRONKITE, E. P. AND BOND, V. P.—(1958) *J. clin. Invest.*, **37**, 904.
JACOBSON, L. O., MARKS, E. K., GASTON, E. O., ROBSON, M. AND ZIRKLE, R. E.—(1949*a*) *Proc. Soc. exp. Biol., N.Y.*, **70**, 740.
*Idem*, MARKS, E. K., ROBSON, M. J., GASTON, E. O. AND ZIRKLE, R. E.—(1949*b*) *J. Lab. clin. Med.*, **34**, 1538.
*Idem* AND SIMMONS, E. L.—(1948) *Anat. Rec.*, **100**, 678.
*Idem*, SIMMONS, E. L., MARKS, E. K., ROBSON, M. J., BETHARD, W. F. AND GASTON, E. O.—(1950) *J. Lab. clin. Med.*, **35**, 746.
KELLY, L. S. AND JONES, H. B.—(1953) *Amer. J. Physiol.*, **172**, 575.
KINDRED, J. E.—(1942) *Amer. J. Anat.*, **71**, 207.
KIRSCHBAUM, A.—(1958) *Blood*, **13**, 1099.
KOHN, H. I. AND FOGH, J. E.—(1959) *J. nat. Cancer Inst.*, **23**, 293.
LAJTHA, L. G.—(1959) Paper read to the Annual Congress of the British Institute of Radiology, 11.xii.59.—(1960*a*) *Brit. J. clin. Path.*, **13**, 275.—(1960*b*) *Brit. J. Radiol.*, **33**, 588.

LAMBERT, G., MAISIN, J., MANDART, M., PLUYGERS, E. AND PODIO, G.—(1950) *C.R. Soc. Biol., Paris*, **144**, 1558.
LEA, D. E. AND GRAY, L. H.—(1956) 'Actions of Radiations on Living Cells'. 2nd Edition. London (Cambridge University Press), p. 74.
LORENZ, E. AND CONGDON, C. C.—(1954) *J. nat. Cancer Inst.*, **14**, 955.
*Idem*, UPHOFF, D., REID, T. R. AND SHELTON, E.—(1951) *Ibid.*, **12**, 197.
MADDOCK, C. L. AND DJERASSI, I.—(1958) *Proc. Amer. Ass. Cancer Res.*, **2**, 323.
MATHÉ, G. AND BERNARD, J.—(1958) *Bull. Ass. franç. Cancer*, **45**, 289.—(1959) *Rev. franç. Ét. clin. Biol.*, **4**, 442.
*Iidem*, DE VRIES, M. J. SCHWARZENBERG, L., LARRIEU, M. J., LALANNE, C. M., DUTREIX, A., AMIEL, J. L. AND SURMONT, J.—(1960) *Rev. Hémat.*, **15**, 115.
MECHANIK, N.—(1926) *Z. Anat. EntwGesch.*, **79**, 58.
MEINEKE, H. A. AND CRAFTS, R. C.—(1956) *Anat. Rec.*, **124**, 47.
MILLER, C. P., HAMMOND, C. W. AND TOMPKINS, M.—(1950*a*) *Science*, **111**, 540.—(1950*b*) *Ibid.*, **111**, 719.—(1951) *J. Lab. clin. Med.*, **38**, 331.
MORKOVIN, D. AND FELDMAN, A.—(1959) *Brit. J. Radiol.*, **32**, 282.—(1960) *Ibid.*, **33**, 197.
NYE, R. N.—(1931) *Proc. Soc. exp. Biol., N.Y.*, **29**, 34.
OEHLBECK, F. W. F., ROBSCHEIT-ROBBINS, F. S. AND WHIPPLE, G. H.—(1932) *J. exp. Med.*, **56**, 425.
OSBORNE, J. W., BRYAN, H. S., QUASTLER, H. AND RHOADES, H. E.—(1952) *Amer. J. Physiol.*, **170**, 414.
OSGOOD, E. E.—(1954) *Blood*, **9**, 1141.
PATT, H. M.—(1957) *Ibid.*, **12**, 777.
*Idem*, MALONEY, M. A. AND JACKSON, E. M.—(1955) *Fed. Proc.*, **14**, 112.
PEGG, D. E. AND KEMP, N. H.—(1960) *Lancet*, ii, 1426.
PUCK, T. T.—(1959) *Rev. mod. Phys.*, **31**, 433.
*Idem* AND MARCUS, P. I.—(1956) *J. exp. Med.*, **103**, 653.
*Idem*, MORKOVIN, D., MARCUS, P. I. AND CIECURA, S. J.—(1957) *Ibid.*, **106**, 485.
REID, T. R. AND GIFFORD, M. P.—(1952) *J. nat. Cancer Inst.*, **13**, 431.
REKERS, P. E. AND COULTER, M.—(1948) *Amer. J. med. Sci.*, **216**, 643.
SARTORELLI, A. C. AND LEPAGE, G. A.—(1957) *Proc. Amer. Ass. Cancer Res.*, **2**, 246.
SCHWARTZ, E. E.—(1958) *Ibid.*, **2**, 343.—(1959) *Acta Radiol., Stockh.*, **52**, 235.
SMITH, W. W., MARSTON, R. Q., GONSHERY, L., ALDERMAN, J. M. AND RUTH, H. J.—(1955) *Amer. J. Physiol.*, **183**, 98.
SPARROW, A. H. AND MIKSCH—(1961) quoted by Z. M. Bacq and P. Alexander in 'Fundamentals of Radiobiology'. 2nd Edition. London (Pergamon Press), p. 308.
SPIERS, F. W.—(1946) *Brit. J. Radiol.*, **19**, 52.—(1949) *Ibid.*, **22**, 521.—(1951) *Ibid.*, **24** 365.
STOHLMAN, F. AND BRECHER, G.—(1956) *Proc. Soc. exp. Biol., N.Y.*, **91**, 1.
*Idem*, CRONKITE, E. P. AND BRECHER, G.—(1955) *Ibid.*, **88**, 402.
SUIT, H. D.—(1957) *Brit. J. clin. Path.*, **10**, 267.
TALBOT, T. R. AND ELSON, L. A.—(1958) *Nature, Lond.*, **181**, 684.
THOMAS, E. D., ASHLEY, C. A., LOCHTE, H. L., JARETZKI, A., SAHLER, O. D. AND FERREBEE, J. W.—(1959) *Blood*, **14**, 720.
TILL, J. E. AND MCCULLOCH, E. A.—(1961) *Radiation Res.*, **14**, 213.
TRAN BA LOC, MATHÉ, G. AND BERNARD, J.—(1958) *Rev. franç. Ét. clin. Biol.*, **3**, 461.
TRENTIN, J. J.—(1957) *Proc. Amer. Ass. Cancer Res.*, **2**, 256.
TUBIANA, M., LALANNE, C. M. AND SURMONT, J.—(1961) *Proc. roy Soc. Med.*, **54**, 1143
UPHOFF, D. E.—(1958) *Blood*, **13**, 1099.
VENDRELY, R.—(1955) 'The Nucleic Acids'. Edited by Chargaff, E. and Davidson, J. N. New York (Academic Press), vol. II, p. 155.
VOGEL, A. W.—(1961) *Cancer Res.*, **21**, 636.
VOS, O., DAVIDS, J. A. G., WEYZEN, W. W. H. AND VAN BEKKUM, D. W.—(1956) *Acta physiol. pharm. néerl.*, **4**, 482.

de Vries, M. J. and Vos, O.—(1958) *J. nat. Cancer Inst.*, **21**, 1117.
Watanabe, S.—(1955) *J. Kyushu hemat. Soc.*, **5**, 132, quoted by Awaya, K., Fujii, H., Tanaka, Y. and Okada, M. (1960) *Arch. Histol. Jap.*, **18**, 473.
Weston, J. K., Maxwell, R. E., Lee, M., Finzel, J. and Fisken, R. A.—(1957) *Fed. Proc.*, **16**, 377.
White, S. G., Logen, J. B., Aggeler, P. M. and Geyer, R. P.—(1953) *Proc. Soc. exp. Biol., N.Y.*, **83**, 384.
Wilson, C. W.—(1950) *Brit. J. Radiol.*, **23**, 92.
Wilson, R. E., Dealy, J. B., Sandowsky, N. L., Corson, J. M. and Murray, J. E.—(1959) *Surgery*, **46**, 261.
Woodard, H. Q. and Holodny, E.—(1960) *Physics in med. Biol.*, **5**, 57.
*Idem* and Spiers, F. W.—(1953) *Brit. J. Radiol.*, **26**, 38.
Yoffey, J. M.—(1961) 'Quantitative Cellular Haematology'. Springfield, Illinois (C. C. Thomas), p. 45.

Since this paper was prepared for publication, I. C. Cree (*Lancet*, 1962, i, 1104) has reported successful bone marrow homografts in rabbits treated with aminochlorambucil.